# Influence of Ellagitannins Extracted by Pomegranate Fruit on Disulfide Isomerase PDIA3 Activity

**DOI:** 10.3390/nu11010186

**Published:** 2019-01-17

**Authors:** Fabio Altieri, Francesco Cairone, Flavia Giamogante, Simone Carradori, Marcello Locatelli, Silvia Chichiarelli, Stefania Cesa

**Affiliations:** 1 Dipartimento di Scienze Biochimiche “A. Rossi Fanelli”, Università degli Studi di Roma “La Sapienza”, Piazzale A. Moro, 5-00185 Rome, Italy; fabio.altieri@uniroma1.it (F.A.); flavia.giamogante@uniroma1.it (F.G.); silvia.chichiarelli@uniroma1.it (S.C.); 2 Istituto Pasteur-Fondazione Cenci Bolognetti, 00161 Roma, Italy; 3 Dipartimento di Chimica e Tecnologie del Farmaco, Università degli Studi di Roma “La Sapienza”, Piazzale Aldo Moro, 5-00185 Roma, Italy; francesco.cairone@uniroma1.it; 4 Dipartimento di Farmacia, Università “G. d’Annunzio” di Chieti-Pescara, Via dei Vestini, 31-66100 Chieti, Italy; marcello.locatelli@unich.it

**Keywords:** pomegranate, punicalagin, ellagic acid, HPLC-DAD, PDIA3, redox activity

## Abstract

Pomegranate fruit is a functional food of high interest for human health due to its wide range of phytochemicals with antioxidant properties are implicated in the prevention of inflammation and cancer. Ellagitannins, such as punicalagin and ellagic acid, play a role as anti-atherogenic and neuroprotective molecules in the complex fighting against the degenerative diseases. The aim of this work was to evaluate the composition in punicalagins and ellagic acid of differently obtained extracts from whole fruit, peels and juices, prepared by squeezing or by centrifugation, of pomegranate belonging to different cultivars. Moreover, a wider phenolic fingerprint was also determined. The bioactivity of the extracts was tested on the redox activity of PDIA3 disulfide isomerase, an enzyme involved in the regulation of several cellular functions and associated with different diseases such as cancer, prion disorders, Alzheimer’s and Parkinson’s diseases. The results demonstrate that the different ratios between punicalagin and ellagic acid modulate the enzyme activity and other ellagitannins could interfere with this activity.

## 1. Introduction

The increasing awareness of the role played by foods and of the relationship between nutrition and health has raised the attention level toward their potential in the prevention of degenerative and chronic ailments. Consumers are increasingly oriented to look for a better lifestyle comprising correct behaviors in dietary choices. It is well known that degenerative illnesses, firstly cardiovascular diseases, represent the leading causes of death in industrialized countries and that diabetes and relative comorbidities are disabling and undermine well-being by lowering life expectancy.

Today’s worldwide dietary guidelines are based on the daily consumption of plant foods rich in phytochemicals able not only to prevent these pathologies but also to maintain a general state of health [1]. Scientific evidence has correlated a high intake of fruits and vegetables rich in phenolic compounds in the protection against cerebrovascular diseases, coronary heart disorders and neurodegenerative diseases [2,3,4]. Polyphenols exert a generic antioxidant and free radical scavenging role next to specific biological properties including anti-inflammatory and anticancer activity modulated by the antiproliferative and antimetastatic effects beyond the regulation of gene expression [5].

Among foods with recognized healthy properties, and belonging to plant-based diet, a dominant role is represented by the pomegranate. Fruits from *Punica granatum* L. represent a typical food of the Mediterranean area, with a tradition historically proven by authoritative ancient sources. Extensively used in the traditional medicine, the three parts of this fruit—the inedible exocarp and mesocarp (peels) and the edible endocarp made by pulp and seeds (arils)—are rich in interesting bioactive molecules, which have been the subjects of many studies in recent years [6,7]. In particular, pomegranate peels represent about the 50% of the whole fruit and are a rich source of phenolics, flavonoids and ellagitannins, such as punicalagins, punicalin, gallagic acid, ellagic acid and relative glycosides [8,9].

Recent clinical studies indicate that pomegranates may improve memory functioning and decrease the risk of ischemic stroke [10] and a recent review reports the many potentials of ellagic acid, which exerts cardiovascular and anti-cancer, anti-obesity, antimicrobial, antiviral and antioxidant activities [11,12]. This small molecule is able to reduce the lipidemic profile and lipid metabolism, modulate pro-inflammatory mediators (tumor necrosis factor-α, interleukin-1β, interleukin-6), and decrease the activity of nuclear factor-κB while increasing nuclear factor 2 erythroid-2 related, playing a pivotal role in anti-atherogenic, anti-inflammatory, and neuroprotective effects [13].

Moreover, among the European Community priorities, the first point is actually represented by “jobs, growth and investment”, and one of the first recognized critical points of these policy areas is the attainment of a circular economy; a goal to reach by helping “European business and consumers to make the transition to a strong and more circular economy, where resources are used in a more sustainable way”. By this point of view, pomegranate peels possess a very high added value, as they represent one of the most valuable by-products of the food industry in terms of ellagitannins [14]. A particularly high punicalagin content (up to about 120 mg/g dry matter) in the pomegranate peels has been reported in the literature [15]. Moreover, peel extracts have recently raised a great interest for their potential use as nutraceuticals or in dietary supplement and, in particular, punicalagin extraction by co-products of pomegranate juice preparation could represent an interesting goal as this polyphenolic component is widely taken into consideration given the wide spectrum of health-promoting activities exerted [16,17]. Despite several phytochemicals found in pomegranate fruits and moreover in peels being recognized as molecules counteracting oxidative stress and preventing some major diseases, their biological targets remain unclear. Then, the investigation of these biological targets could open a new research field in order to clarify the phytochemicals biological mechanisms.

In this regard, it has been repeatedly reported that the protein disulfide isomerase 3 (PDIA3) can be modulated by several types of polyphenols in different pathological conditions [18,19]. In vitro studies have shown that PDIA3 can interact with several macromolecules and small ligands, such as green tea catechins and flavonoids [20,21,22]. More recently, it has been demonstrated that punicalagin, alone as a single and pure component, can bind to PDIA3 and is a non-competitive inhibitor of PDIA3 reductase activity with an inhibition constant within the micromolar range [23]. PDIA3 is a protein mainly localized into the endoplasmic reticulum but, differently to others PDIs, PDIA3 is also present on the cellular surface and in other cellular compartments [24] and hence represents an attractive biological target for natural compounds.

PDIA3 is also involved in several cellular functions and consequently associated with different human diseases such as cancer, prion disorders, Alzheimer’s and Parkinson’s diseases [25,26,27,28]. Its structure is characterized by four thioredoxin-like domains, namely a, b, b’ and a’. The a and a’ domains contain the catalytic active sites constituted by the tetrapeptide Cys-Gly-His-Cys, which provides PDIA3 with redox activity, while b and b’ domains are redox inactive but are required for the PDIA3 complete activity [24,29].

Furthermore, PDIA3 is present on the platelet surface [30], where it is involved in platelet aggregation due to its redox activity towards the β3 integrin, a subunit of the fibrinogen receptor αIIbβ3, and induces a new pattern of disulfide bond formation. In fact, under thrombin stimulation, the fibrinogen receptor αIIbβ3 on the platelets surface requires several conformational changes to drive platelet activation [31,32,33,34]. Since the role of PDIA3 in platelet aggregation is well-known, many researchers are focusing on its possible inhibition [35]. Thus, the search of PDIA3 modulators could be interesting and pomegranate extracts can be a good source of these compounds.

In light of these interesting fields, the rationale of this work was to recover and valorize the co-products of agro-industrial processes, with the final goal of obtaining interesting biomolecules to use as pharmaceutics or with nutritional, nutraceutical and cosmetic health purposes. Indeed, this work provides information to confirm and extend the already obtained data exploiting the properties of the pomegranate fruit peel that generally represents a waste of pomegranate juice preparation, even though it contains significant amounts of phenolic bioactive compounds.

To achieve such goal, we focused our efforts on realizing different extracts from diversely worked-up pomegranates, optimizing the extraction procedure of the bioactive compounds of interest in order not only to obtain extracts enriched in polyphenols but also characterized by a different peculiar composition and finally, evaluating their effects as possible PDIA3 modulators. With this aim, we selected different pomegranate fruits coming from several Italian districts and belonging to different cultivars which were submitted to different work-up and extraction techniques. The resulting extracts, evaluated in their quali-quantitative composition by two different HPLC-DAD analyses, were then tested for their biological activity in terms of inhibition effect on PDIA3 reductase activity.

## 2. Materials and Methods

### 2.1. Materials

Bidistilled water, ethanol, 85% formic acid, ethyl acetate, glacial acetic acid, dimethyl sulfoxide (DMSO), acetonitrile RS for HPLC, punicalagin (≥98%), ellagic acid (≥95%), phosphate buffered saline (PBS), dithiothreitol (DTT), oxidized glutathione (GSSG) and eosin isothiocyanate were purchased from Sigma (Milan, Italy). EDTA (0.5 M solution pH 8.0) from IBI Scientific (Dubuque, Iowa).

Pomegranate fruits of cv. Mollar (Ml) were obtained from an Italian market. Some fruits of Italian origin, belonging to two sub-varieties of cv. Dente di Cavallo (DC_1_ and DC_2_) with a difference of about two weeks in the maturation stage and harvest time, were provided by a local supplier (Azienda Biologica Giovomel, Avellino, Italy). A pomegranate variety from Mozia island (South Italy) was provided by the “Missione Archeologica Mozia” of “Sapienza” University of Rome (Mz).

### 2.2. Samples Preparation

Whole fruits (*F*) or separated into two parts: arils and peels (exocarp and mesocarp) were washed, mixed for 30 s and submitted to the extraction procedure. Peels (*P*), manually separated from washed fruits, were blended in a mixer for 30 s and submitted to the extraction procedure. Juices were prepared from whole fruit by squeezing (*S*) or from arils by centrifugation (*C*).

### 2.3. Extraction Methods

#### 2.3.1. Whole Fruit or Peels Extraction with Ethanol and Acidified Water

All samples (*F* and *P*) were submitted to the extraction procedure as previously described in Reference [7]. In brief, 10 g was blended and extracted with 40 mL ethanol:acidified water (5% acetic acid) in 3:1 (*v:v*) ratio by stirring for 24 h at room temperature in the dark. The extraction mixture was decanted, filtered on paper, evaporated at 40 °C in the dark under vacuum and immediately analyzed or stored at 4 °C.

#### 2.3.2. Juice Extraction with Ethanol

Squeezed or centrifuged juice (2 mL) was submitted to extraction with ethanol (6 mL) under stirring at room temperature in the dark for 24 h. The extraction mixture, decanted and filtered on paper, was evaporated at 40 °C in the dark under vacuum and immediately analyzed or stored at 4 °C.

#### 2.3.3. Partitioning in Ethyl Acetate

The so-obtained dried extracts, dissolved in 10 mL of water, were extracted thrice in a separating funnel with 10 mL of ethyl acetate, freeze-dried or evaporated at 40 °C under vacuum in the dark. The samples were immediately analyzed or stored at 4 °C.

### 2.4. HPLC-DAD Analyses

Dried extracts were weighed, dissolved in DMSO or water and filtered before injection into an HPLC Perkin Elmer apparatus (Waltham, MA, USA) consisting of a Series 200 LC pump, a Series 200 DAD and a Series 200 autosampler, including a Totalchrom Perkin Elmer software for the data acquisition. Chromatography was performed on a Luna RP18 column (250 × 4.6 mm i.d., 5 μm) using a mobile phase made by acetonitrile and water acidified by 5% formic acid, in gradient with a flow rate of 1 mL/min, at 360 and 530 nm. Punicalagin anomers α and β and ellagic acid were quantified as previously described [7]. The multi-component phenolic fingerprint was also studied for the most promising pomegranate extracts (DC and Mz) according to a validated method as reported in the Supplementary Materials [36].

### 2.5. PDIA3 Purification and Disulfide Reductase Activity Determination

#### 2.5.1. Protein Expression and Purification

Human recombinant PDIA3 was cloned into a pET21 vector (Novagen) and expressed in *E. coli* strain BL21 as previously described [37]. Protein was purified by ammonium sulphate fractionation, ion exchange and heparin chromatography [38,39]. Protein purification was evaluated by SDS-PAGE and its concentration was spectrophotometrically determined (ε_280_ reduced form = 44,810 M^−1^·cm^−1^).

#### 2.5.2. Determination of Protein Disulfide Reductase Activity

Disulfide reductase activity of PDIA3 was evaluated by sensitive fluorescent assay using dieosin glutathione disulfide (DiE-GSSG) as a fluorogenic probe. DiE-GSSG was synthesized by the reaction of eosin isothiocyanate with oxidized glutathione [40] with some modifications [20]. DiE-GSSG purification was carried out by HPLC and its concentration was determined spectrophotometrically (ε_525_ = 88,000 M^−1^·cm^−1^). A sample from each dried pomegranate extract (50 µL), from the peel and whole fruit of the different cultivars (Ml, Mz, DC_1_ and DC_2_, respectively), was dissolved in 100 µL of ultrapure water at a final concentration of 500 mg/mL. Their effects on PDIA3 disulfide reductase activity were evaluated, using DiE-GSSG 200 nM, PDIA3 20 nM, DTT 5 µM in reaction buffer (PBS and EDTA 0.2 mM). All tested extracts were tested at final concentrations of 666.0, 66.0, 6.60 and 0.66 µg/mL with a two-minute incubation before the analysis. GSSG reduction was monitored for 3 min at 545 nm, with excitation at 520 nm, at 25 °C under continuous stirring. Enzymatic activity was evaluated by fitting the slope of the initial fluorescence increase. The percentage of the inhibition was expressed as a residual activity in presence of the extracts compared to PDIA3 alone. IC_50_ was evaluated by fitting the inhibition effects vs. extracts concentration using the GraphPad Prism 7.0 software (GraphPad Software, San Diego, CA, USA).

### 2.6. Statistical Analysis

Each assay was replicated at least three times. Data are expressed as mean ± SEM and statistical significance was determined using the GraphPad Prism 7.0 software (GraphPad Software, San Diego, CA, USA).

## 3. Results

### 3.1. Polyphenols Extraction

Fully ripe pomegranate fruits coming from different geographic origins and belonging to several cultivars selected as previously described in the section “Material and methods” (Mz, Ml, DC_1_, DC_2_), were supplied. These were characterized by different organoleptic quality (color and size, flavor, harvest dates, peel texture and thickness), and were submitted to different work-up with the aim to obtain products with diverse ellagitannin composition. Whole fruits were homogenized (*F*) or squeezed (*S*); separated peels were homogenized (*P*), separated arils were centrifuged (*C*). The resulting homogenates and juices were submitted to a mild extraction method, by a simple ethanol/water mixture, which grants a high yield in polyphenolic extraction, as previously reported [7]. All of the samples were submitted to hydroalcoholic extraction which yields range among 8–19% by dry weight in whole fruit, most likely in relation to the not evaluated sugar content but are not directly correlated to the polyphenolic content, as shown below by HPLC analyses. The range, only apparently similar for the peels (12–19%), assumes a different means because, in this case, sugars are almost completely absent. On the contrary, the extraction yields from juices showed a narrow range between 15–16% mainly accounting for the sugar content of the edible part.

The dried extracts obtained from these experiments were then extracted with a partitioning system made of water and ethyl acetate, which allows a preferential distribution of punicalagin in the aqueous phase and of ellagic acid, much less represented, in the organic phase of medium polarity. The extraction yields account for the partitioning of about 95% in the aqueous fraction and only a small part in the organic phase that accounts for a maximum of 6.2% in the sample *P*-DC_2_.

### 3.2. Quali-Quantitative Analysis of the Polyphenolic Fraction

**Figure 1:**
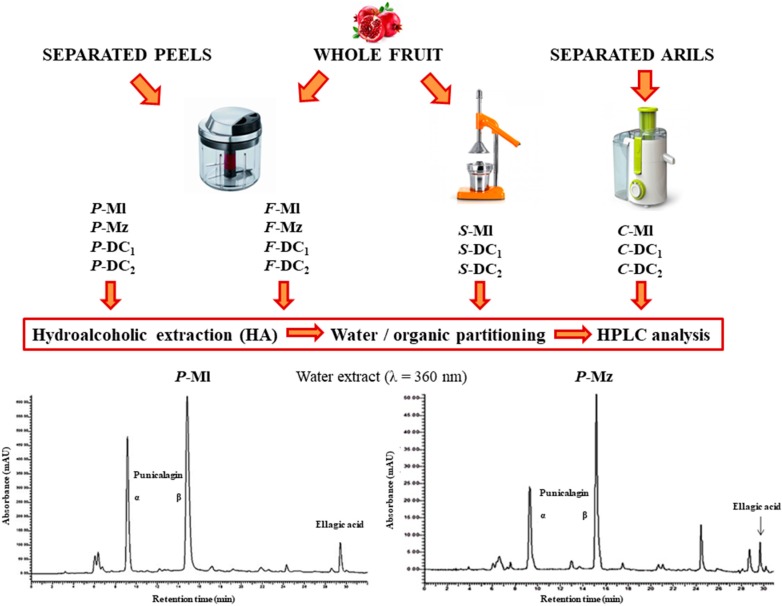
Flow chart and chromatographic profile of the phenolic compounds of selected samples.

The variability shown by the samples was taken into account, in terms of extraction yields of hydroalcoholic extracts, is highly significant in regards to the differences existing among geographic provenance, cultivars and even sub-varieties grown in the same geographic and pedoclimatic conditions but only the quali-quantitative profile obtained by HPLC-DAD analyses could show the real differences in terms of polyphenolics, and specifically for ellagitannins. Important differences could already be revealed by a simple glance to the chromatograms of the hydroalcoholic extracts (Figure 1) recorded at 360 nm, which show the dominant presence of punicalagin α and β anomers, followed by a lower ellagic content but also, in some cases (as for example in the Mz sample), of other non-negligible molecules.

The quantification of punicalagins as sum of anomers, reported as mg/g by dry extract (Table 1), evidenced relevant differences, in the range 5–16 mg/g dry aqueous extracts by fruit, 16–30 mg/g of aqueous extracts by peels and in amounts around or much less than 1 mg in the aqueous extracts by squeezed or centrifuged juices. In addition, if the values were very low, significant differences could be shown among the two kinds of applied work-up. Juices obtained by squeezing of the whole fruit show punicalagin and ellagic acid contents of about 17- and 2-fold respect to juices obtained by centrifugation of separated arils. The ellagic acid is concentrated in the organic phase in which it could reach contents of 37 mg/g dry extract (*P*-Mz). Taking into account the overall quantity of the extracts, we could evaluate the content of punicalagin ranging between 150 and 500 mg/100 g and of ellagic acid ranging between 15 and 65 mg/100 g of fresh homogenized peels which largely justify the employment of this matrix for the extraction of such a valuable moiety. Indeed, punicalagin seems to reach a high concentration in the organic phase, in which only the ellagitannin fraction is selectively extracted, but the 73.5 mg/g dry extract (*P*-Ml) of the richest sample correspond, taking into account the extract yield of 0.36%, only to an absolute quantity of about 25 mg/100 g fresh homogenized peels.

To further analyze the presence of other important phytochemicals in these extracts, we carried out an analysis of the multi-component phenolic pattern by a validated HPLC method. We looked for twenty-two secondary metabolites abundant in the plant kingdom. Surprisingly, few of them were detected and quantitated in some extracts. Pomegranate peel was richer in phenolic compounds than the whole fruit (~2-fold for Mz and ~11-fold for DC_1_ samples). Conversely, we found an opposite profile in *F*-DC_2_. The most characterizing compounds were rutin and catechin. Moreover, 3-hydroxy-4-methoxybenzaldehyde was only present in *P*-DC_1_, whereas *P*-Mz showed small amounts of 2,3-dimethoxy benzoic acid and benzoic acid. Finally, *F*-Mz was characterized by the presence of vanillic acid (Tables S1 and S2, Supplementary Materials). An important aspect to be considered is that both pomegranate treatments (centrifugation and squeezing) led to a complete loss of these secondary metabolites.

### 3.3. Pomegranate Extract Effects on Disulfide Redox Activity of the PDIA3

**Figure 2:**
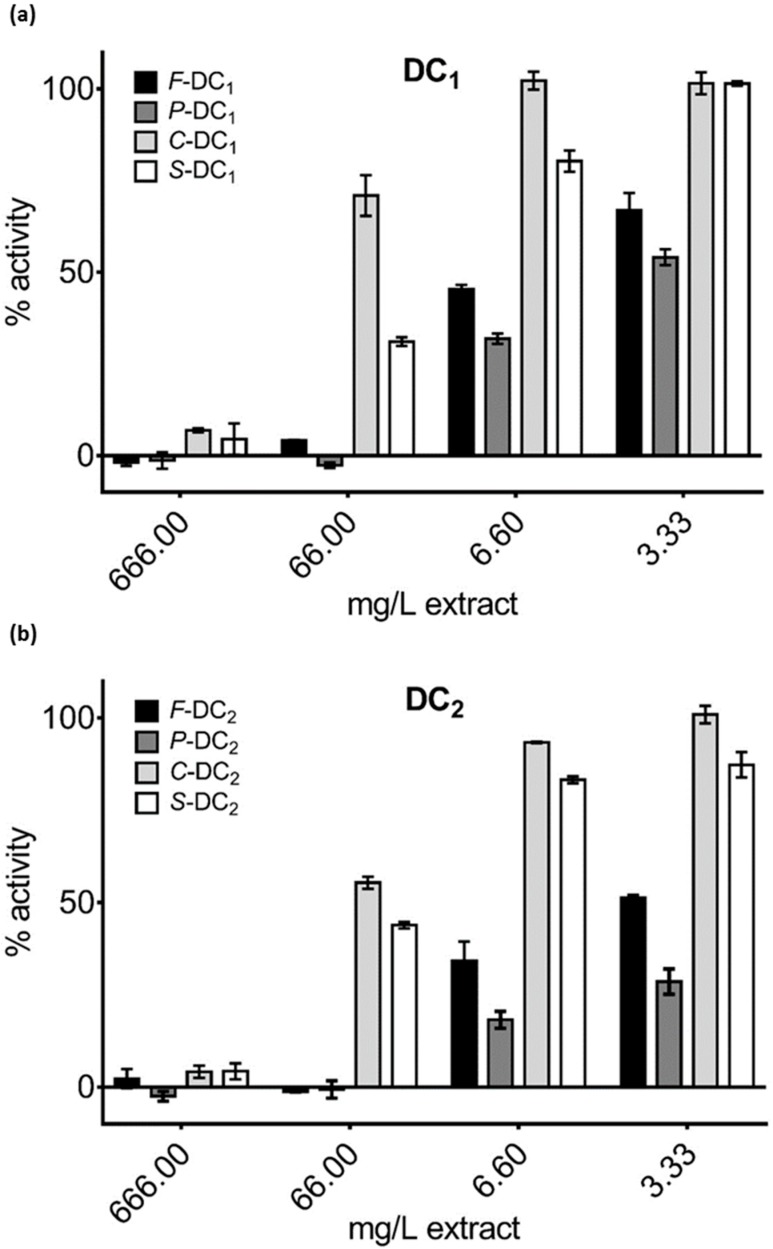
Residual activity of protein disulfide isomerase 3 (PDIA3) in the presence of (**a**) DC_1_ and (**b**) DC_2_ extracts.

**Figure 3:**
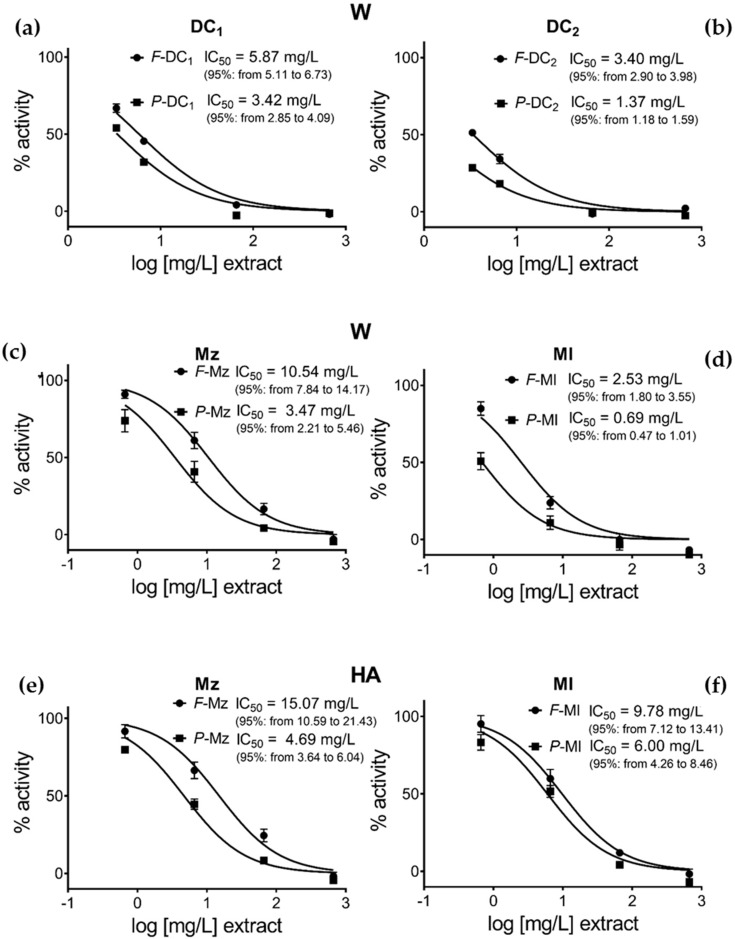
Comparison of the effects of the different obtained extracts on PDIA3 activity: (**a**) DC_1_ aqueous extract; (**b**) DC_2_ aqueous extract; (**c**) Mz aqueous extract; (**d**) Ml aqueous extract; (**e**) Mz hydroalcoholic extract; (**f**) Ml hydroalcoholic extract.

Pomegranate extracts by whole fruit, separated peels, squeezed and centrifuged juices from samples DC_1_ and DC_2_ were tested on PDIA3 redox activity. Decreasing amounts of selected extracts were added to the reaction mixture solution, to a final concentration ranging from 666 to 3.33 µg/mL, and PDIA3 residual activity was assayed. Generally, as shown in Figure 2, only peel and whole fruit aqueous extracts were able to significantly inhibit the PDIA3 redox activity in a dose-dependent manner, while juices, either prepared by squeezing or centrifugation, show inhibition only at the highest concentrations. For this reason, we extended the analysis only to peel and whole fruit extracts of the other selected samples; testing their activity at a concentration ranging from 666 to 0.66 µg/mL (Figure 3). Aqueous peel extracts, *P*-Ml, *P*-Mz, *P*-DC_1_ and *P*-DC_2_ always resulted in a higher capacity of PDIA3 inhibition compared to their corresponding aqueous whole fruit extracts, *F*-Ml, *F*-Mz, *F*-DC_1_ and *F*-DC_2_ respectively, even though at a higher concentration, completely inhibit the protein activity (Figure 3, confirming the high potency of Mollar cultivar respect to the other ones (Figure 3d).

Other interesting reported evidence is the higher activity of Ml and Mz aqueous extracts compared to the corresponding hydroalcoholic ones (Figure 3c–f), with a more evident difference in Mollar cultivar. In addition, the results from the hydroalcoholic extracts confirm that the separated peels appear more active than the whole fruit as already shown for aqueous extracts (Figure 3e,f).

In Table 1 is the reported punicalagin concentration and in Table 2 the ratio punicalagin/ellagic acid is compared to the effects on PDIA3 redox activity of the tested samples. From the data, it can be observed that the different inhibitory activity of each extract is highly correlated to its punicalagin concentration, suggesting that punicalagin is the main component responsible for PDIA3 inhibition. Considering that the IC_50_ value of pure punicalagin is within the micromolar range [23], the activity of this molecule seems not greatly affected when it is tested in a mixture with other ellagitannin components. On the contrary, assuming that the observed inhibitory effect is just related to punicalagin, the IC_50_ calculated on the basis of punicalagin content of each extract tested is always below 0.1 μM, strongly supporting a synergic effect between punicalagin and other components present in the extract.

## 4. Discussion

Given the rising interest for the biological properties exerted by pomegranate ellagitannin component and the related healthy aspects, the aim of this work was to evaluate the application of a simple and mild extraction procedure to a matrix which can provide a high added value. The recovery and valorization of the co-products of agro-industrial processes have the double advantage to recycle waste, reducing the global impact, and to obtain interesting biomolecules to use as pharmaceutics or with nutritional, nutraceutical and cosmetic healthy purposes [41]. Peels generally represent a waste of pomegranate juice preparation, while containing significant amounts of phenolic bioactive compounds, not only with antioxidant properties but also able to modulate protein activity as well as the disulphide redox activity of the PDIA3, an interesting biological target.

In the present work, the possibility to obtain extracts containing several amounts of differently purified ellagitannins represents a crucial starting point in the evaluation of the phytocomplex capacity to interfere with biological activity, assessing in which way the different extract composition could influence the protein activity. A very higher amount of ellagitannins was found, as expected, in the peels respect to the whole fruit, but also differently work-up juices were analyzed with the aim to highlight that simple domestic practices, such as fruit squeezing or arils centrifugation are, could deeply influence the final content of biomolecules in a home-made beverage. The extraction of homogenized whole fruit represents a medium content among the other evaluated samples and accounts for all the different extractable components. The use of safe reagents, such as water, ethanol and acetic acid, the easiness of processing and the reduced procedural costs, make the performed work-up directly applied for the preparation of food supplements. The partitioning in water, essential in our experimental design, to obtain differently distributed ellagitannin, may imply an expensive freeze drying or an undesired spray drying step, detrimental to thermosensible molecules, but these could be easily avoided if the aim is obtaining food supplements. The partitioning in ethyl acetate is necessary only if ellagic acid should be more selectively extracted and concentrated, and although considered acceptable for safe extraction techniques, this solvent should be removed very carefully before human use. For this reason and also because of a response of less than the 10% of total extracted punicalagin, the organic phase was not tested in the biological assays. It could become of interest only in view of evaluating further the different ratios between punicalagin and ellagic acid, the last one becoming comparable or preeminent in the acetate fraction.

As previously shown, in some cultivars, punicalagin rises to amounts of 400–500 mg/100 g fresh peels, representing an exceptionally high content of specific polyphenols such as ellagitannins are. In the preparations obtained by peels, the ratio between punicalagin and ellagic acid varies in a very wide range between 75:1 (*P*-Ml) in the aqueous extracts and 1:1 in the organic ones (*P*-Mz), up to organic extracts from juices in which punicalagin is almost absent.

Both peel and whole fruit extracts are able to inhibit the PDIA3 redox activity in a dose-dependent manner. The reported higher capacity on PDIA3 inhibition of the peel extracts could be due to the higher punicalagin content respect to the corresponding whole fruit, supporting the hypothesis that the inhibitory effect is mainly correlated to punicalagin. Indeed, analyzing the effect on PDIA3 activity of both punicalagin and ellagic acid, alone as single and pure components, previously published data showed a higher inhibitory effect of punicalagin compared to ellagic acid, although both molecules can bind to PDIA3 with a not so high different affinity [23]. Hence, the present analysis carried out on different pomegranate extracts, allowed us to test the effect of punicalagin and ellagic acid on PDIA3 activity directly from a natural mixture, evaluating the synergic or antagonist activity of these two molecules which can be present in different ratios. In addition, this analysis allowed also to speculate about the contribution of other species present into the extracts, besides punicalagin, in modulating PDIA3 redox activity inhibitory.

Extracts of the same kind, obtained by a hydroalcoholic (HA) extraction system or after partitioning of these in water (W), showed a different activity with the HA always less effective respect to W. This is more evident in *P*-Ml, where peels extracted by W present a higher PDIA3 inhibition activity respect to the HA (Table 2). This behavior could be correlated to the different punicalagin content but also to the different punicalagin/ellagic acid ratio that can be associated with different analytes distribution after partitioning in water (or in ethyl acetate). Indeed, the punicalagin/ellagic acid ratio drastically increases in aqueous extract of the *P*-Ml (75:1) compared to corresponding hydroalcoholic extract (32:1). Additionally, the aqueous *P*-Ml extract which shows the maximum PDIA3 inhibition effect with an IC_50_ around 0.69 µg/mL, has the highest punicalagin/ellagic ratio.

A synergic effect may be expected since the inhibitory activity of the tested extracts seems always higher than it would be expected considering only the punicalagin content. On the basis of data reported in Table 2, a hypothetical value of IC_50_ can be calculated by assuming that punicalagin is mainly responsible for the observed inhibitory effect and this is solely the result of the amount of punicalagin present in each fraction tested. Considering that the IC_50_ value previously calculated for punicalagin as the isolated component is within the micromolar range [23], the activity of this molecule seems not negatively affected when it is present in a complex mixture with other components. On the contrary, for all analyzed extracts, these hypothetical IC_50_ values were always smaller than the value of IC_50_ previously calculated for punicalagin alone. This observation strongly supports a synergistic effect due to the presence of other components in the analyzed extracts that are responsible for the better inhibitory activity observed. Analyzing data reported in Table 2, water extract (W), further considerations could be made. Ml extracts always appear more active than Mz ones, regardless of the type of extract (whole fruit or peels). However, both Ml and Mz extracts show a similar ratio of about 3 between the IC_50_ (Extract, mg/L) of whole fruit and of peel (*F*-Ml/*P*-Ml = 2.53/0.69 and *F*-Mz/*P*-Mz = 10.54/3.48) demonstrating the higher activity of peel components, mostly related to the higher content of punicalagin. When these extracts are compared for the hypothetical IC_50_, (Punic µg/L, amount of punicalagin present in each extract), this ratio decreases to about 2 and 1.2, respectively (IC_50_ µM Punic *F*-Ml/*P*-Ml = 0.037/0.019 and *F*-Mz/*P*-Mz = 0.101/0.080) indicating a similar synergistic effect, with peel extracts appearing always more active than whole fruit ones and Ml cultivar extracts showing a better inhibitory effect.

A different behavior can be instead observed for DC cultivar belonging to different sub-varieties, (DC_1_ and DC_2_). Although the inhibitory effect of the peel extracts is always better than whole fruit, DC_2_ shows a stronger activity than DC_1_ even if the *P-*DC_2_ extract is characterized by a lower content of punicalagin. Comparing the IC_50_ of both DC_2_ extracts (*F* and *P*) with the Ml ones, DC_2_ extracts seems to be less active. However, the hypothetical IC_50_ calculated on the basis of punicalagin content of both *P*-DC_2_ and *F*-DC_2_ are quite similar to the corresponding hypothetical IC_50_ calculated for the *P-*Ml extract. So, while these data still support a synergism in the inhibitory effect due to the presence of other components in the extracts, this effect is not the same among the analyzed cultivars, probably as a result of a different composition. Additionally, for DC cultivar the sub-variety may play an important role, affecting both the extract composition and punicalagin content as well as the observed inhibitory effect.

Besides, all the considered aqueous extracts at 66 µg punicalagin/mL, inhibit almost completely the protein activity with the exception of *F*-Mz, where a 30% residual activity is still observable. Thus, Mz whole fruit extracts seem to be less effective in PDIA3 inhibition. Although *F*-DC_2_ and *F*-Mz have similar punicalagin content and the same punicalagin/ellagic acid ratio, 30:1, they differ in PDIA3 inhibition activity. The *F*-Mz shows a different chromatographic profile in which several peaks not present in other analyzed cultivars are detectable. From literature [42] four reported peaks, identified as punicalagin-derivative, punicalagin-derivative, HHDP-hexoside and ellagic acid-*O*-pentoside, could interfere in PDIA3 inhibition. Then, further studies will be necessary in order to better investigate the effects of pomegranate components on PDIA3 activity and evaluate the activity of extracts obtained from different cultivar or maturation stage.

Many studies suggest that PDIA3 is involved in multiple cellular functions through its specific enzymatic activity and its wide binding capabilities, and these functions are not confined within the endoplasmic reticulum. Moreover, considering the role of PDIA3 in several types of diseases, it could become an important target for developing novel therapeutic strategies. Thus, the finding of strong and specific PDIA3 inhibitors could open a new field in the treatment of these diseases and this study opens the possibility to use pomegranate extract as a modulator of PDIA3 activity.

## 5. Conclusions

In an eco-sustainable context, a very simple hydroalcoholic extraction allows us to transform by-products of pomegranate juice industry in interesting co-products with high added value. In our opinion, the choice of this matrix and the biocompatible and economic adopted extraction technique could be of high interest for the scientific community and for the food supplement industry.

We here report evidence that pomegranate extracts can inhibit/modulate the reductase activity of PDIA3 as previously demonstrated for punicalagin. Considering the many cellular processes involving PDIA3, healthy uses of pomegranate extracts deserve to be evaluated. In the light of the recent reports showing that PDIA3 plays an important role in platelet aggregation, sperm maturation and egg fertilization as its redox and chaperone activities of PDIA3 were important for these functions, the reported inhibition effects of the differently obtained pomegranate extracts could be also useful for future drug application and their relative development.

## Figures and Tables

**Table 1:** Quali–quantitative analyses of the two studied metabolites in the differently obtained pomegranate extracts.

Sample	Hydroalcoholic (HA)		Water (W)		Organic	
	Punicalagin	Ellagic Acid	Punicalagin	Ellagic Acid	Punicalagin	Ellagic Acid
*F*-DC_1_	-	-	5.13 ± 0.11	0.07 ± 0.02	-	-
*P*-DC_1_	-	-	25.9 ± 0.02	0.76 ± 0.07	-	-
*C*-DC_1_	-	-	0.05 ± 0.02	0.006 ± 0.003	-	-
*S*-DC_1_	-	-	1.26 ± 0.10	0.017 ± 0.003	-	-
*F*-DC_2_	-	-	6.53 ± 0.09	0.22 ± 0.09	-	-
*P*-DC_2_	-	-	16.2 ± 0.01	0.80 ± 0.04	-	-
*C*-DC_2_	-	-	0.06 ± 0.04	0.007 ± 0.004	-	-
*S*-DC_2_	-	-	0.72 ± 0.05	0.008 ± 0.004	-	-
*F-*Mz	11.2 ± 0.11	1.57 ± 0.02	10.4 ± 0.12	0.34 ± 0.08	36.7 ± 0.20	24.6 ± 0.13
*P*-Mz	25.7 ± 0.24	3.40 ± 0.19	24.9 ± 0.05	1.08 ± 0.13	35.1 ± 0.14	37.4 ± 0.05
*F-*Ml	19.1 ± 0.20	0.59 ± 0.03	15.8 ± 0.15	0.32 ± 0.07	54.8 ± 0.26	14.7 ± 0.09
*P*-Ml	32.2 ± 0.35	1.00 ± 0.25	30.3 ± 0.17	0.40 ± 0.08	73.5 ± 0.58	17.8 ± 0.06
*C*-Ml	0.06 ± 0.03	0.012 ± 0.003	0.05 ± 0.01	0.007 ± 0.002	BLD *	2.45 ± 0.50
*S*-Ml	0.85 ± 0.45	0.026 ± 0.007	0.79 ± 0.07	0.01 ± 0.009	BLD *	1.25 ± 0.30

* BLD: Below Limit of Detection.

**Table 2:** Punicalagin relative effects on PDIA3 in hydroalcoholic (HA) and water (W) extracts after partitioning.

Sample	Hydroalcoholic (HA)				Water (W)			
	From Table 1		Calculated		From Table 1		Calculated	
	Pun/extr mg/g	IC_50_ extr mg/L	IC_50_ Pun µg/L	IC_50_ Pun µM	Pun/extr mg/g	IC_50_ extr mg/L	IC_50_ Pun µg/L	IC_50_ Pun µM
	A	B	AxB	AxB/MW	A	B	AxB	AxB/MW
*F*-Mz	11.2	15.07	168.8	0.156	10.4	10.54	109.2	0.101
*P*-Mz	25.7	4.69	120.3	0.111	24.9	3.48	86.7	0.080
*F*-Ml	19.1	9.78	186.8	0.172	15.8	2.53	40.0	0.037
*P*-Ml	32.2	6.00	193.2	0.178	30.3	0.69	20.9	0.019
*F*-DC_1_	-	-	-	-	5.13	5.87	30.2	0.028
*P*-DC_1_	-	-	-	-	25.88	3.42	88.6	0.082
*F*-DC_2_	-	-	-	-	6.53	3.4	22.2	0.020
*P*-DC_2_	-	-	-	-	16.14	1.37	22.2	0.020

## Supplementary Materials

The following are available online at http://www.mdpi.com/2072-6643/11/1/186/s1, HPLC-DAD method and equipment were described along with the quali-quantitative composition of the most promising extracts (DC and Mz) in terms of specific phenolic compounds. Table S1. Multi-component phenolic pattern of DC cultivars. Table S2. Multi-component phenolic pattern of Mz cultivar.
