# Supplementary material for: Influence of Ellagitannins Extracted by Pomegranate Fruit on Disulfide Isomerase PDIA3 Activity
Source: Nutrients. 2019 Jan 17;11(1):186. doi: 10.3390/nu11010186 (PMC6356990; doi:10.3390/nu11010186)

Supplementary Materials

Influence of Ellagitannins Extracted by Pomegranate Fruit on Disulfide Isomerase PDIA3 Activity

Fabio Altieri [1,4], Francesco Cairone [2], Flavia Giamogante [1], Simone Carradori [3,*], Marcello Locatelli [3], Silvia Chichiarelli [1] and Stefania Cesa [2,*]

[1] Dipartimento di Scienze Biochimiche "A. Rossi Fanelli", Sapienza University ", Piazzale A. Moro 5, 00185, Rome, Italy; fabio.altieri@uniroma1.it (F.A.), flavia.giamogante@uniroma1.it (F.G.), silvia.chichiarelli@uniroma1.it (S.Ch.)
[2] Dipartimento di Chimica e Tecnologie del Farmaco, Università degli Studi di Roma "La Sapienza", Piazzale Aldo Moro, 5 - 00185 Roma, Italy; francesco.cairone@uniroma1.it (F.C.), stefania.cesa@uniroma1.it (S.Ce.)
[3] Dipartimento di Farmacia, Università "G. d'Annunzio" di Chieti-Pescara, Via dei Vestini, 31 - 66100 Chieti, Italy ; simone.carradori@unich.it (S.C.); marcello.locatelli@unich.it (M.L.)
[4] Istituto Pasteur-Fondazione Cenci Bolognetti, Italy
* Correspondence: simone.carradori@unich.it; Tel.: +39-0871-3554583 (S.C.), stefania.cesa@uniroma1.it; Tel.: + 39-06-49913198; fax: + 39-06-49913133 (S.Ce.)

Materials

Chemical standards: gallic acid, catechin, chlorogenic acid, 4-hydroxybenzoic acid, vanillic acid, epicatechin, syringic acid, 3-hydroxybenzoic acid, 3-hydroxy-4-methoxybenzaldehyde, *p*-coumaric acid, rutin, sinapinic acid, *t*-ferulic acid, naringin, 2,3-dimethoxybenzoic acid, benzoic acid, *o*-coumaric acid, quercetin, 8-cinnamoyl harpagide (harpagoside), *t*-cinnamic acid, naringenin, and carvacrol (> 98%) were purchased from Sigma-Aldrich (Milan, Italy). Solvents (acetonitrile (HPLC-grade), methanol (HPLC-grade), acetic acid (≥ 99%)) were obtained from Carlo Erba Reagents (Milan, Italy). Centrifuge model 5804 (Eppendorf, Hamburg, Germany), vortex (VELP Scientifica Srl, Usmate, Italy), and ultrasound bath (Falc Instruments, Treviglio, Italy) were used as additional equipment.

HPLC analysis

HPLC-PDA analyses were performed using an HPLC Waters liquid chromatography (model 600 solvent pump, 2996 PDA). Mobile phase was directly degassed *on-line* using a Biotech 4CH DEGASI Compact (Onsala, Sweden). Empower v.2 Software (Waters Spa, Milford, MA, USA) was used to collect and analyze data. The analyses were carried out using gradient elution mode on a C18 reversed-phase column (Prodigy ODS(3), 4.6 x 150 mm, 5 µm; Phenomenex, Torrance, CA), thermostated at 30 °C (± 1 °C). The gradient elution was achieved by a solution of water-acetonitrile (93:7 ratio, with 3% of acetic acid) as initial conditions, and the complete separation was achieved in 60 min. Chemical standards chromatogram (@ 278 nm as example of wavelength in which all compounds show absorbance) for the analytes, with a table reporting the retention

times and the maximum wavelengths used for the quantitative analyses. A table with gradient elution program used for the analyses was also reported.

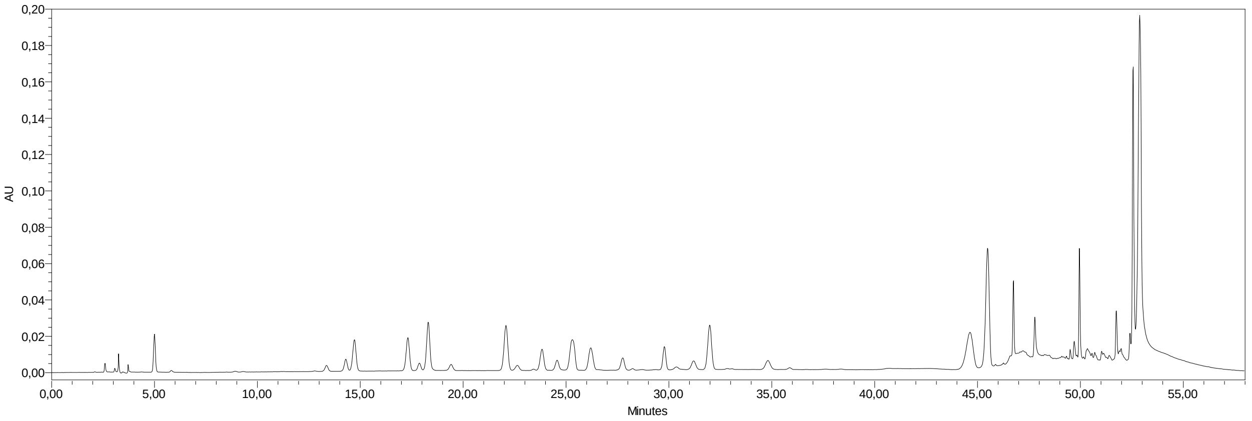

Gradient elution:

**TIME (min)**	**FLOW (mL min^{-1})**	**%A**	**%B**
0		93	7
0.1		93	7
30		72	28
38	1	75	25
45		2	98
47		2	98
48		93	7
58		93	7

Analytes, retention times, and maximum wavelengths used for quantitative analyses:

**Analytes**	**Retention times (min)**	**λ max**
Gallic acid	4.99	271 nm
Catechin	13.36	278 nm
Chlorogenic acid	14.29	324 nm
4-hydroxybenzoic acid	14.71	256 nm
Vanillic acid	17.31	260 nm
Epicatechin	18.30	278 nm
Syringic acid	18.50	274 nm
3-hydroxybenzoic acid	19.41	275 nm
3-hydroxy-4-methoxybenzaldehyde	22.08	278 nm
p-coumaric acid	22.65	310 nm
Rutin	25.38	256 nm
Sinapinic acid	26.18	324 nm
t-ferulic acid	27.75	315 nm
Naringin	29.78	285 nm
2,3-dimethoxybenzoic acid	30.36	299 nm
Benzoic acid	31.20	275 nm
o-coumaric acid	34.81	276 nm
Quercetin	40.57	367 nm
8-cinnamoyl harpagide	45.49	280 nm
t-cinnamic acid	45.87	276 nm
Naringenin	46.74	290 nm
Carvacrol	49.95	275 nm

In the following tables are reported the results in terms of μg/mg. In the tables are reported standard deviations of three independent measurements.

Table S1. Multi-component phenolic pattern of DC cultivars.

CONCENTRATION (µg/mg)	*F*-DC_1	*C*-DC_1	*S*-DC_1	*P*-DC_1	*F*-DC_2	*C*-DC_2	*S*-DC_2	*P*-DC_2
Gallic acid								
Catechin					4.2±0.3			
Chlorogenic acid								
p-OH benzoic acid								
Vanillic acid								
Epicatechin								
Syringic acid								
3-OH benzoic acid				BLQ				
3-OH-4-MeO benzaldehyde				3.5±0.1				
p-coumaric acid								
Rutin	1.66±0.14		BLD	14.3±1.4	5.1±0.2			
Sinapinic acid								
t-ferulic acid								
Naringin								
2.3-diMeO benzoic acid								
Benzoic acid								
o-coumaric acid								
Quercetin								
Harpagoside								
t-cinnamic acid								
Naringenin								
Carvacrol								
**Total (µg/mg)**	**1.66**	**0.00**	**0.00**	**17.75**	**9.23**	**0.00**	**0.00**	**0.00**

Table S2. Multi-component phenolic pattern of Mz cultivar.

CONCENTRATION (µg/mg)	*F*-Mz	*P*-Mz
Gallic acid		
Catechin	2.8±0.3	5.0±0.1
Chlorogenic acid		
p-OH benzoic acid		
Vanillic acid	0.32±0.03	
Epicatechin		
Syringic acid		
3-OH benzoic acid		
3-OH-4-MeO benzaldehyde		
p-coumaric acid		
Rutin	3.8±0.2	6.1±0.6
Sinapinic acid		
t-ferulic acid		
Naringin		
2.3-diMeO benzoic acid		0.56±0.06
Benzoic acid		0.30±0.02
o-coumaric acid		
Quercetin		
Harpagoside		
t-cinnamic acid		
Naringenin		
Carvacrol		
**Total (µg/mg)**	**6.92**	**11.97**

F-DC$_1$ (@278 nm as example of wavelength in which all compounds show absorbance)

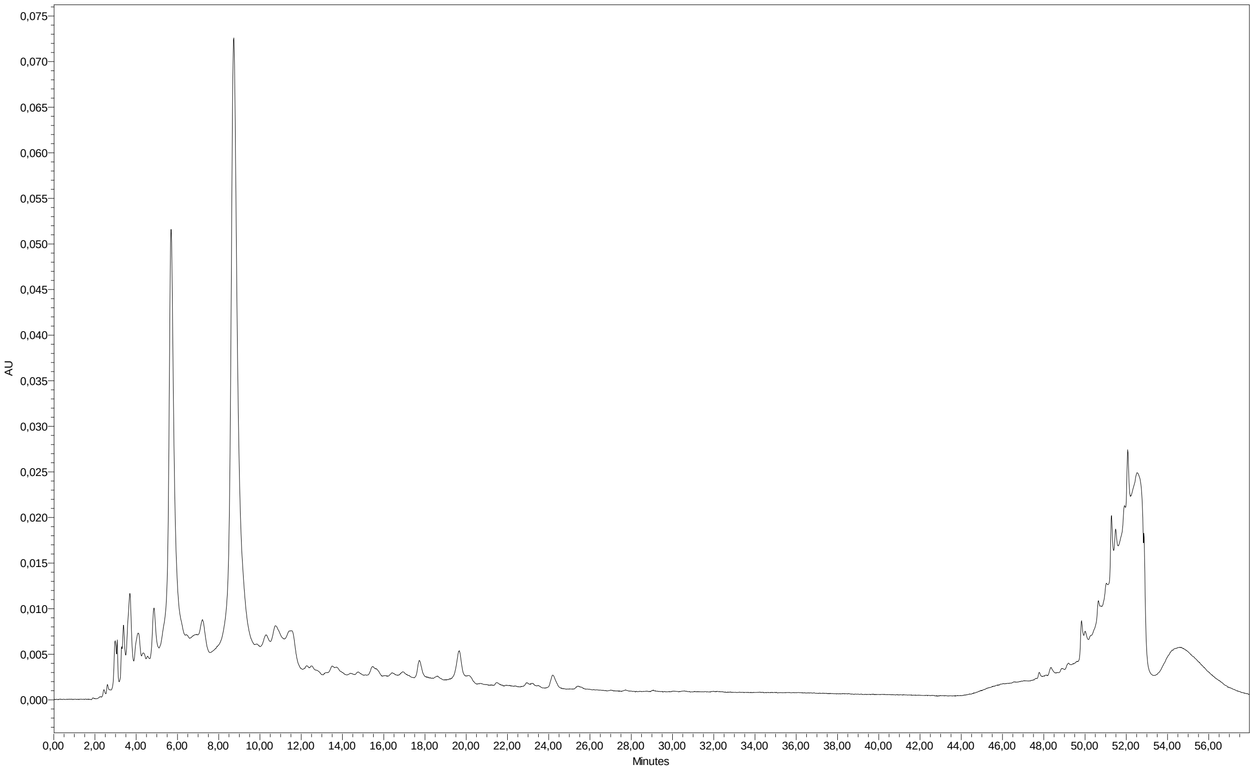

C-DC$_1$ (@278 nm as example of wavelength in which all compounds show absorbance)

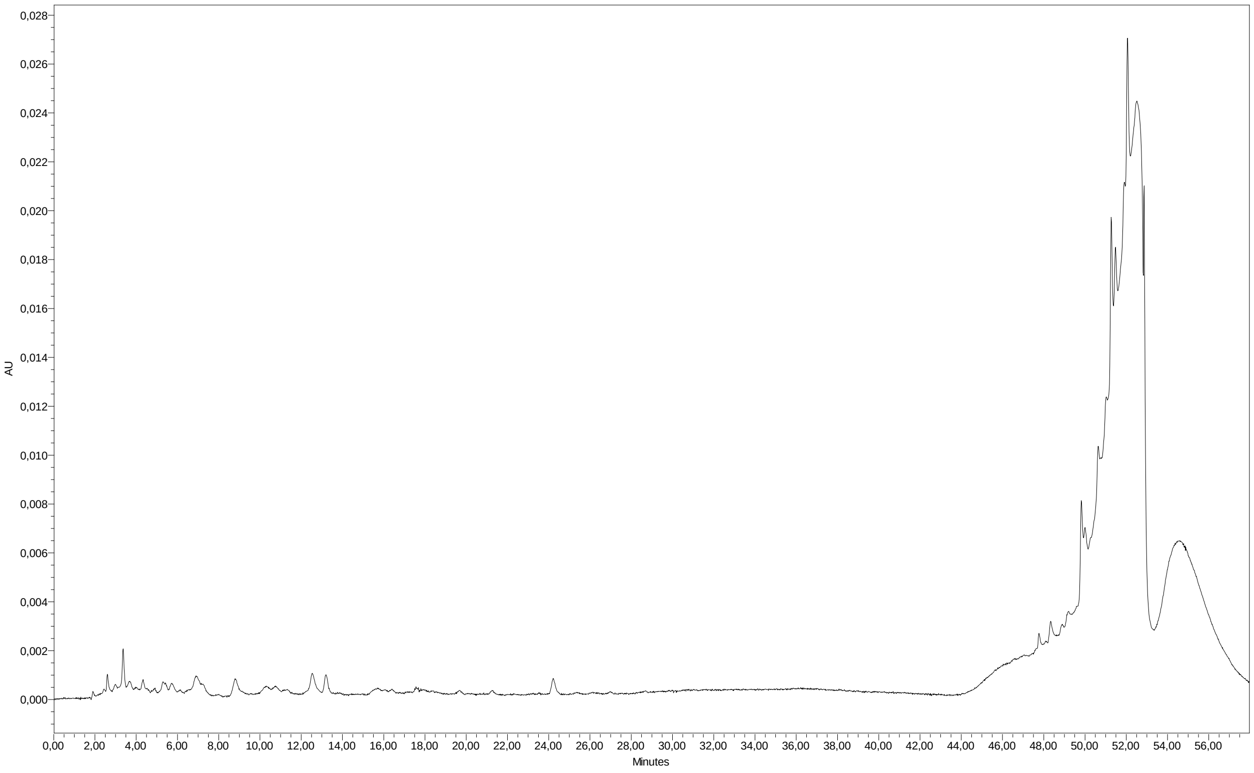

S-DC_1 (@278 nm as example of wavelength in which all compounds show absorbance)

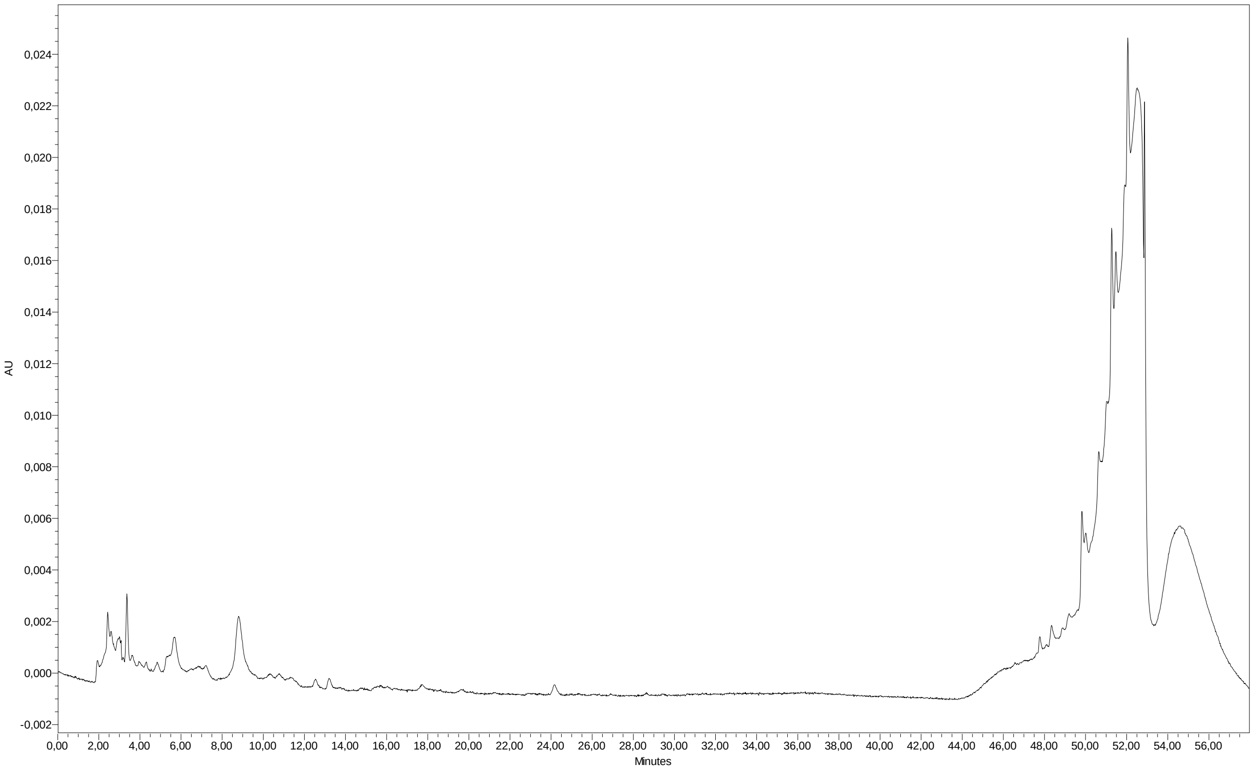

P-DC_1 (@278 nm as example of wavelength in which all compounds show absorbance)

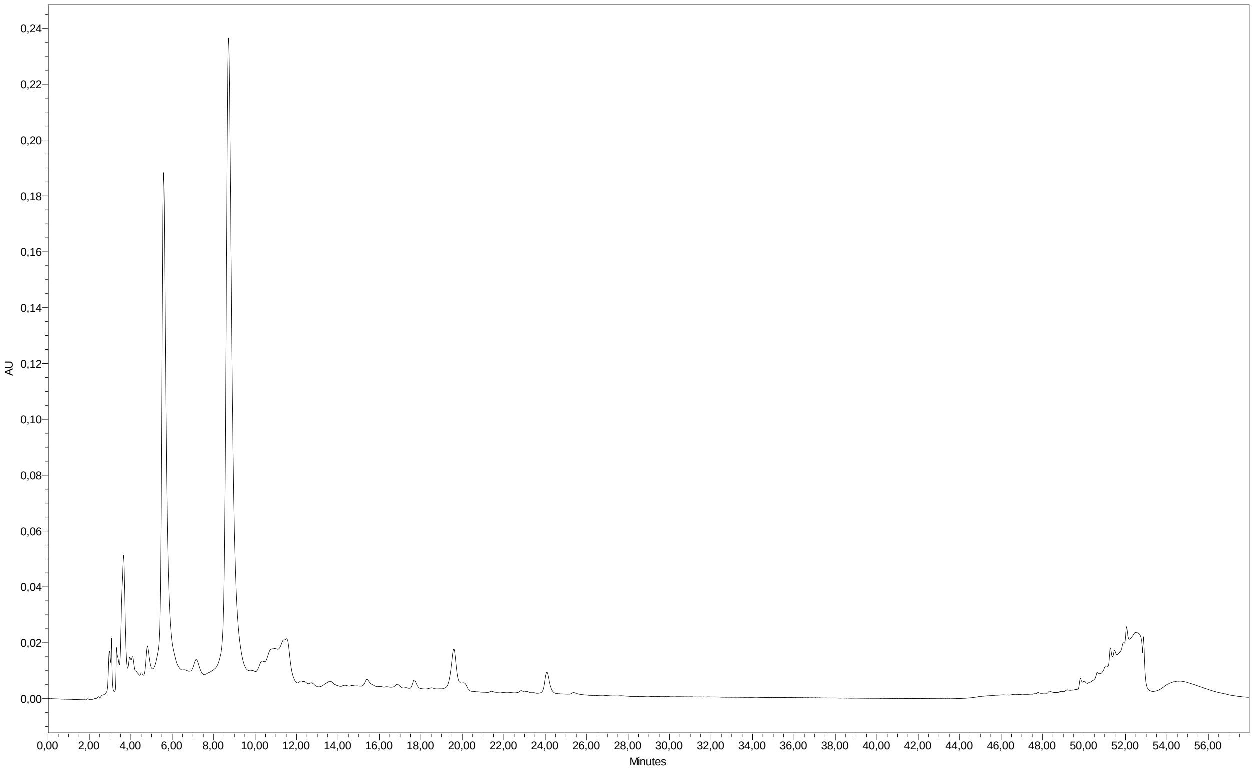

F-DC_2 (@278 nm as example of wavelength in which all compounds show absorbance)

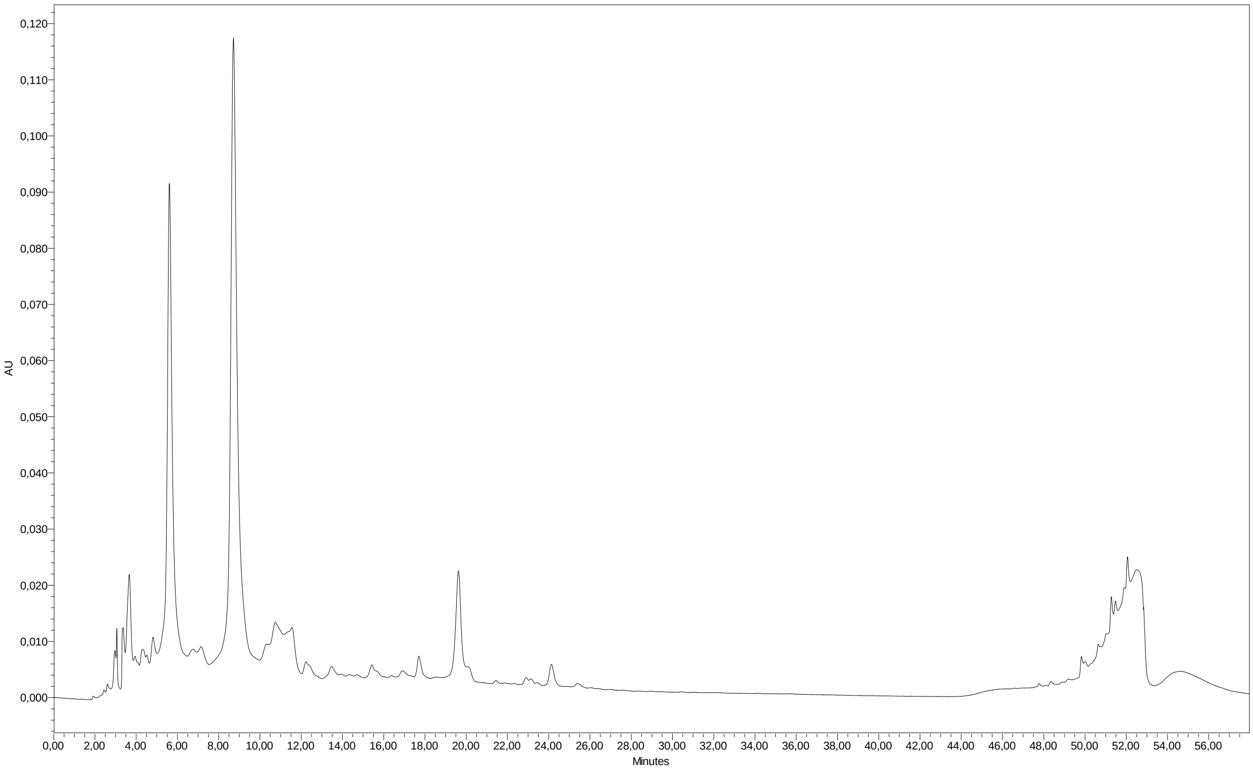

C-DC_2 (@278 nm as example of wavelength in which all compounds show absorbance)

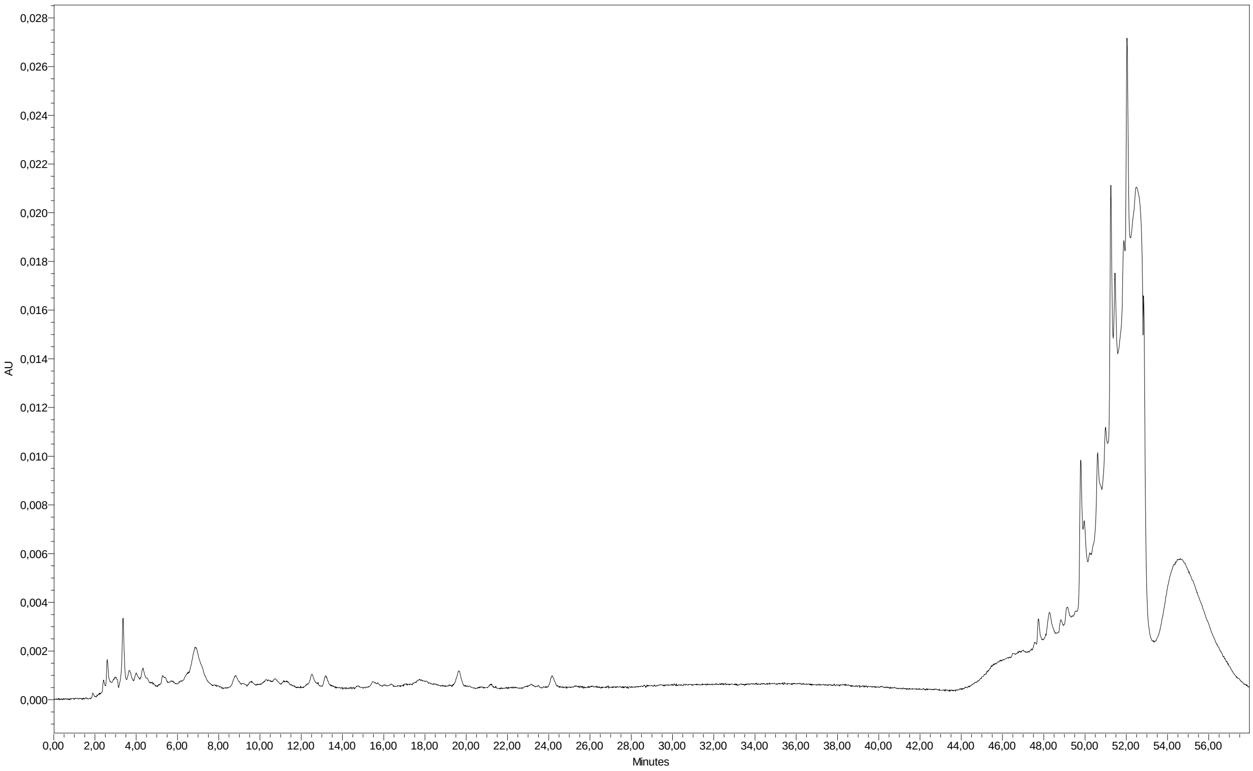

S-DC2 (@278 nm as example of wavelength in which all compounds show absorbance)

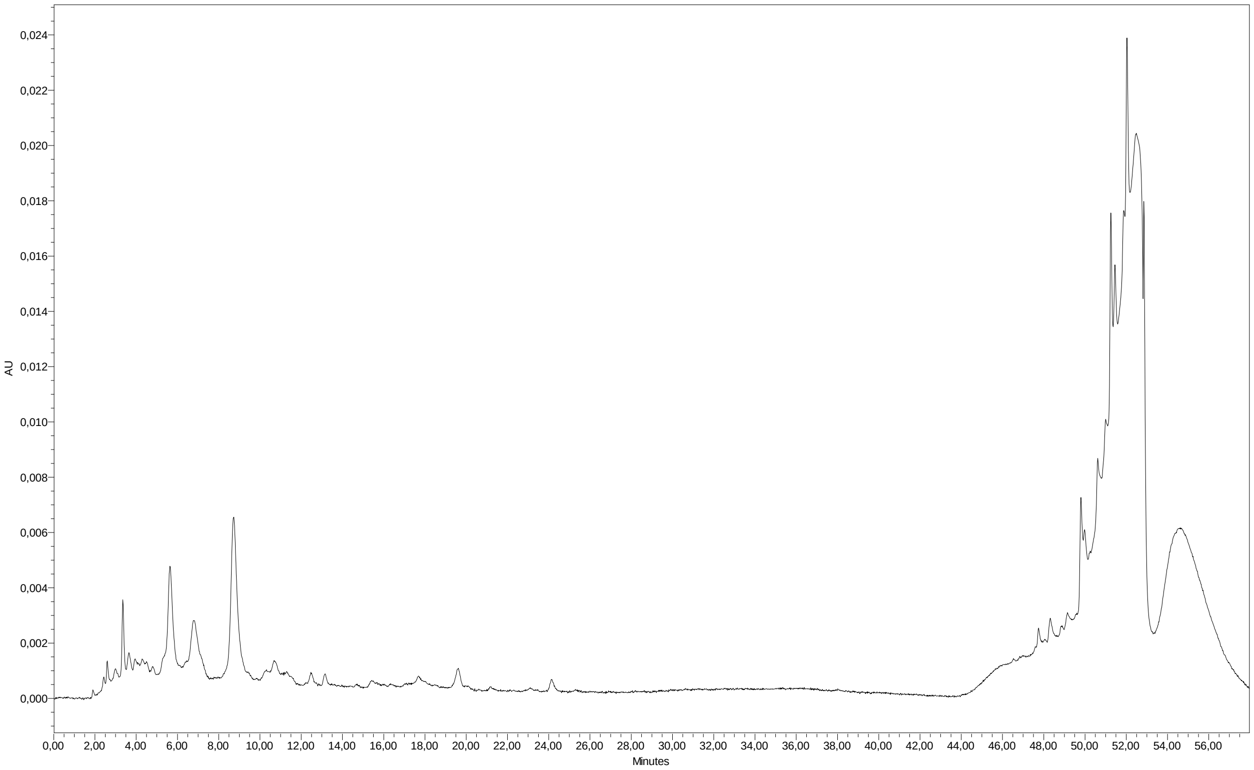

P-DC2 (@278 nm as example of wavelength in which all compounds show absorbance)

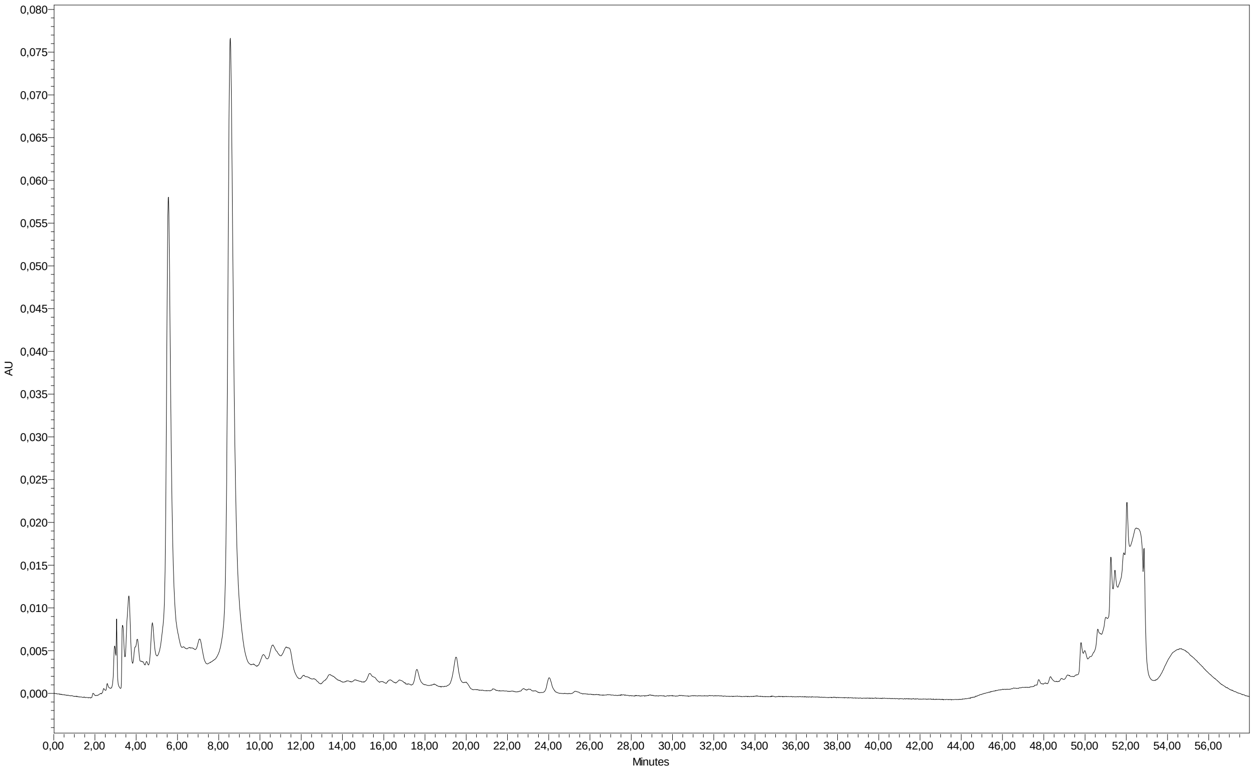

F-Mz (@278 nm as example of wavelength in which all compounds show absorbance)

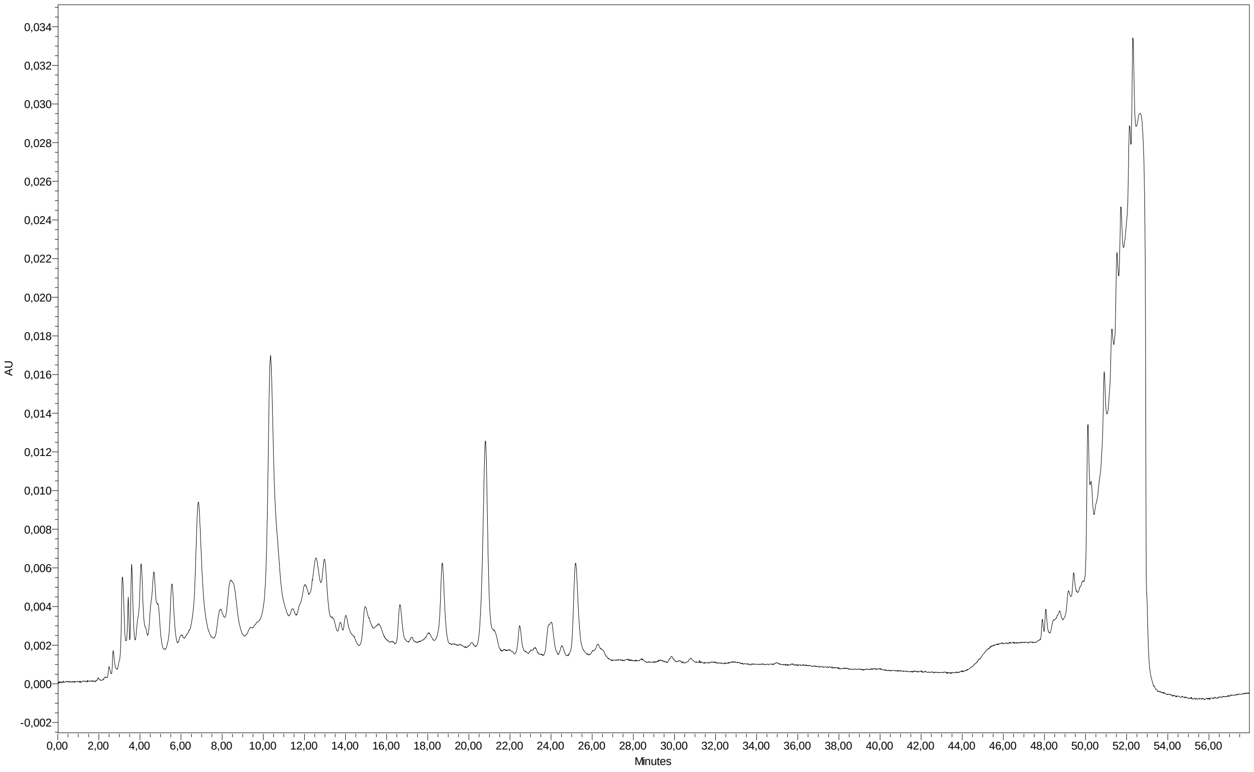

P-Mz (@278 nm as example of wavelength in which all compounds show absorbance)

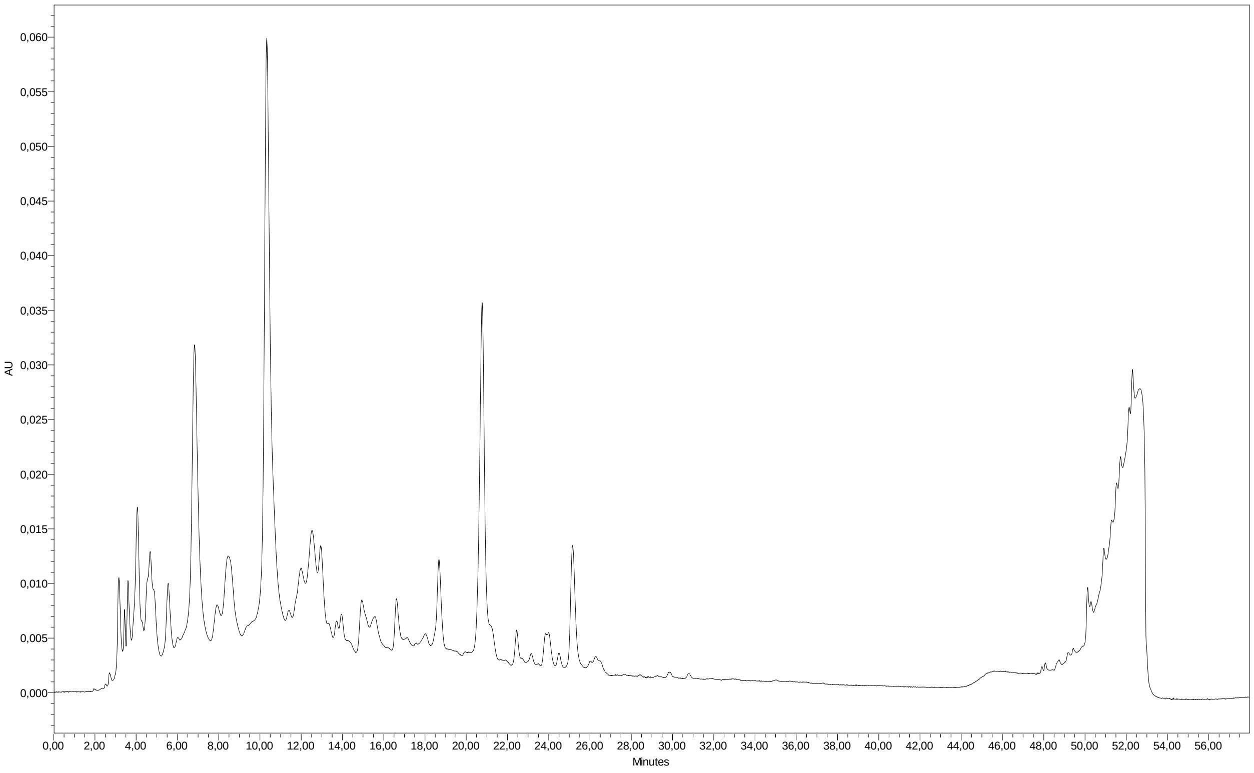

Supplement: Supplementary file 1 [file nutrients-11-00186-s001.pdf]
